# Fick versus flow: a real-time invasive cardiovascular magnetic resonance (iCMR) reproducibility study

**DOI:** 10.1186/s12968-021-00784-7

**Published:** 2021-07-19

**Authors:** Yousef Arar, Tarique Hussain, Riad Abou Zahr, Vasu Gooty, Joshua S. Greer, Rong Huang, Jennifer Hernandez, Jamie King, Gerald Greil, Surendranath R. Veeram Reddy

**Affiliations:** 1 Department of Pediatrics, University of Texas Southwestern Medical Center, Dallas, TX USA; 2 Department of Radiology, University of Texas Southwestern Medical Center, Dallas, TX USA; 3 Pediatric Cardiology, Children’s Medical Center, 1935 Medical District Drive, Dallas, TX 75235 USA; 4 Research Administration, Children’s Medical Center, Dallas, TX USA; 5 Anesthesiology and Pain Management, Children’s Medical Center, Dallas, TX USA

**Keywords:** Congenital heart disease, Interventional CMR, Cardiac catheterization, Magnetic resonance imaging, Device tracking, Reproducibility

## Abstract

**Background:**

Cardiac catheterization and cardiovascular magnetic resonance (CMR) imaging have distinct diagnostic roles in the congenital heart disease (CHD) population. Invasive CMR (iCMR) allows for a more thorough assessment of cardiac hemodynamics at the same time under the same conditions. It is assumed but not proven that iCMR gives an incremental value by providing more accurate flow quantification.

**Methods:**

Subjects with CHD underwent real-time 1.5 T iCMR using a passive catheter tracking technique with partial saturation pulse of 40° to visualize the gadolinium-filled balloon, CMR-conditional guidewire, and cardiac structures simultaneously to aid in completion of right (RHC) and left heart catheterization (LHC). Repeat iCMR and catheterization measurements were performed to compare reliability by the Pearson (PCC) and concordance correlation coefficients (CCC).

**Results:**

Thirty CHD (20 single ventricle and 10 bi-ventricular) subjects with a median age and weight of 8.3 years (2–33) and 27.7 kg (9.2–80), respectively,  successfully underwent iCMR RHC and LHC. No catheter related complications were encountered. Time taken for first pass RHC and LHC/aortic pull back was 5.1, and 2.9 min, respectively. Total success rate to obtain required data points to complete Fick principle calculations for all patients was 321/328 (98%). One patient with multiple shunts was an outlier and excluded from further analysis. The PCC for catheter-derived pulmonary blood flow (Qp) (0.89, p < 0.001) is slightly lower than iCMR-derived Qp (0.96, p < 0.001), whereas catheter-derived systemic blood flow (Qs) (0.62, p = < 0.001) was considerably lower than iCMR-derived Qs (0.94, p < 0.001). CCC agreement for Qp at baseline (C1-CCC = 0.65, 95% CI 0.41–0.81) and retested conditions (C2-CCC = 0.78, 95% CI 0.58–0.89) were better than for Qs at baseline (C1-CCC = 0.22, 95% CI − 0.15–0.53) and retested conditions (C2-CCC = 0.52, 95% CI 0.17–0.76).

**Conclusion:**

This study further validates hemodynamic measurements obtained via iCMR. iCMR-derived flows have considerably higher test–retest reliability for Qs. iCMR evaluations allow for more reproducible hemodynamic assessments in the CHD population.

## Background

Non-invasive cardiovascular magnetic resonance (CMR) and invasive cardiac catheterization have distinct diagnostic roles in the congenital heart disease (CHD) population [1, 2]. CMR contributes important volume and flow data while cardiac catheterization allows for direct pressure and saturation measurements. By linking these powerful modalities, invasive CMR (iCMR) allows for a more accurate and thorough assessment of cardiac hemodynamics at the same time under the same conditions. Accurate pulmonary vascular resistance (PVR) and systemic blood flow measurements are critical for clinical decision making.

In this study, we describe a hemodynamics reproducibility comparison of catheter-based Fick principle measurements (equations outlined below) and CMR-derived pulmonary (Qp) and systemic (Qs) blood flows. Fick principle calculations require measurement of oxygen consumption (VO_2_) and hemoglobin (Hg), as well as obtaining blood saturations in specific locations throughout the body (pulmonary veins = PV, pulmonary artery = PA, mixed venous = MV). PVR and systemic vascular resistance (SVR) can then be determined by obtaining pressure measurements across the pulmonary bed (transpulmonary pressure gradient (TPG)) and systemic circulation (transsystemic pressure gradient (TSG)).

$$Qp = \frac{VO2}{{13.6 x Hg x \left( {PV - PA Sat} \right)}} Qs = \frac{VO2}{{13.6 x Hg x \left( {systemic - MV Sat} \right)}}\quad PVR = \frac{TPG}{{Qp}} SVR = \frac{TSG}{{Qs}}$$

All catheter-based measurements were performed under real-time CMR imaging to guide a gadolinium-filled balloon and an MR-conditional guidewire without the use of ionizing radiation.

## Methods

### Study population 

The protocol was approved by the Institutional Review Board (IRB) (STU 032017-061) and was performed in the Children’s Health CMR catheterization suite at Children’s Medical Center (CMC) in Dallas, Texas, USA. In this protocol, we conducted heart catheterization using real-time CMR in pediatric and adult subjects already undergoing clinically indicated heart catheterization for CHD. In many subjects, CMR was also clinically indicated. If CMR was not required for clinical purposes, only CMR function and flow measurements were acquired as a research procedure. After discussing the indications for and risks of the procedure, prospective informed consent and assent were obtained from all subjects and/or legal guardians as appropriate for all study related procedures.

### iCMR environment and equipment (Fig. 1)

**Fig. 1:**
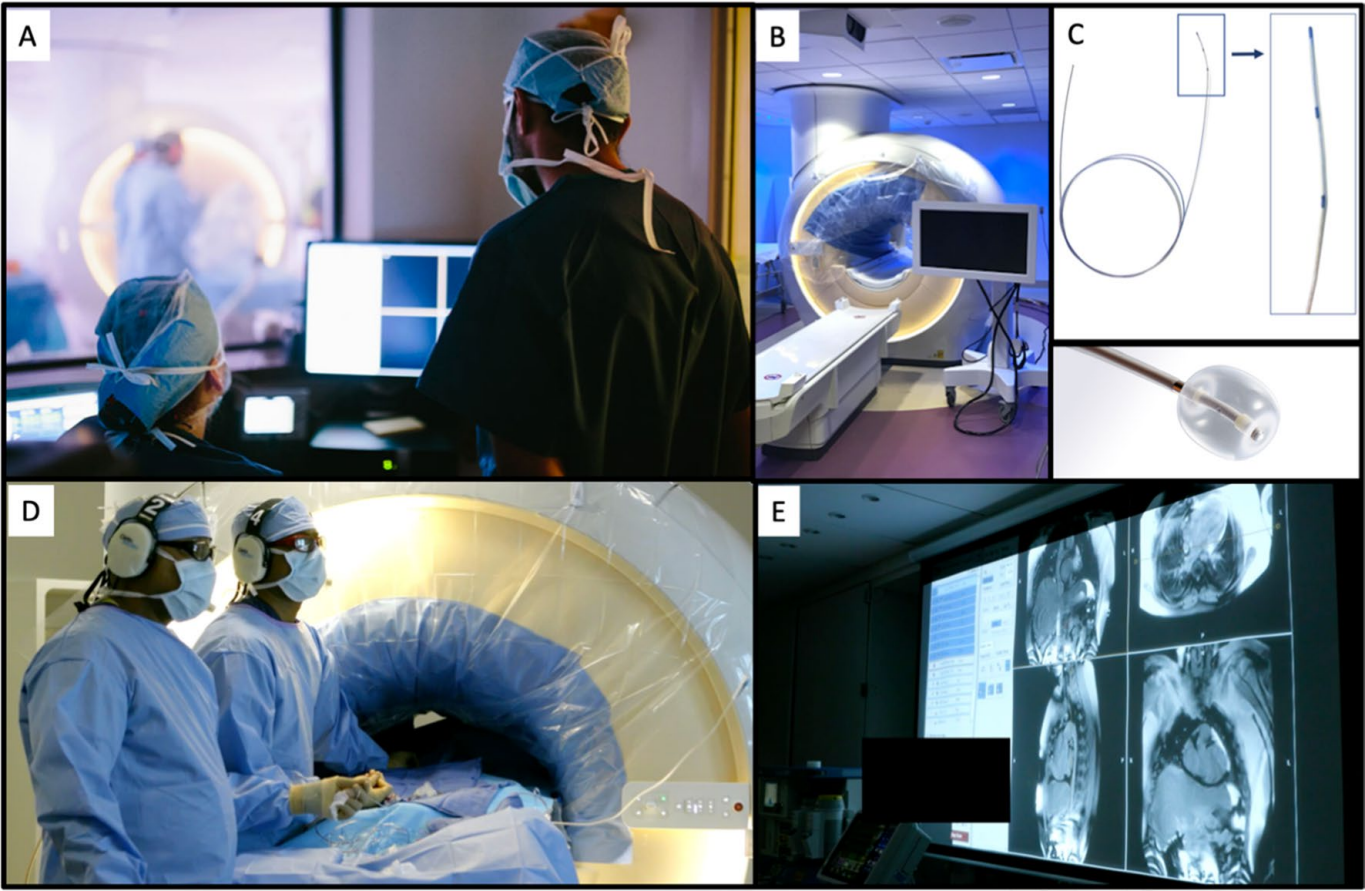
Invasive cardiovascular magnetic resonance (iCMR) environment and equipment. A CMR team (zone 3) adjusting images with direct visualization of the interventionalist performing the iCMR procedure. B Depiction of sterile draping within the iCMR environment. C Interventionalist equipment includes an MR-conditional catheter and guidewire. The FDA cleared and CE marked guidewire has three passive markers, coated with nanoparticles, that produces a distinct susceptibility artifact (0 mm, 20 mm, and 40 mm from the tip). D Interventionalist performing an iCMR procedure (zone 4) with real-time CMR guidance on adjacent projector screen (E)

Our iCMR environment and equipment has been previously described in detail [3]. Briefly, the interventionalist has direct visualization of catheter-derived pressures via the bedside PRiMEGen system [National Institutes of Health, Bethesda, Maryland, USA] [4] during the iCMR procedure. This system feeds into a standard hemodynamic recording system (Sensis, Siemens Healthineers, Erlangen, Germany). This recording, together with the real-time CMR images, is projected in-room by using a shielded projector system [standard projector system for in-room Ambient system ambiance (Philips Healthcare, Best, the Netherlands)] (Fig. 1E).

### MR-conditional catheter and guidewire (Fig. 1C)

A 6-French Arrow, Balloon Wedge-Pressure Catheter (Model AI-07124 and/or AI-07126, Teleflex, Wayne, Pennsylvania, USA) was used for all procedures. The balloon-tip of the catheter was filled with dilute gadolinium (1-part gadolinium to 99-parts saline) and guided with the help of an MR-conditional guidewire to specific structures in the heart to obtain necessary hemodynamics. This 0.035″ guidewire is United States Food and Drug Administration (FDA) cleared and Conformité Européenne (CE) marked MR-conditional guidewire (angled-tip Emeryglide MRWire, Nano4Imaging, Aachen, Germany). Left heart catheterization (LHC) was performed with a 4-French balloon wedge or a 4-French non-braided pigtail catheter (Soft-Vu, Angiodynamics, Latham, New York, USA). The femoral artery sheath was upsized to 6-French only when the MR-conditional wire was necessary.

### Interventionalist timeout and access (zone 3)

Once anesthesia induction is complete, the groin and/or neck are sterilely prepared and draped. The iCMR team performs a separate timeout. A baseline VO_2_ is measured and recorded for catheter-based Fick method calculations. If the subject was < 15 kg, VO_2_ was assumed based on published references [5]. Three patients included in this study were < 15 kg at the time of their procedure. Percutaneous entry with short sheaths are placed in the femoral vein (6-French), femoral artery (4-French), and, if necessary, internal jugular vein (6-French) with ultrasound guidance. The 4-French sheath was upsized to a 6-French sheath whenever necessary to accommodate the 0.035″ MR-conditional guidewire. At this time, an initial blood gas and activated clotting time were drawn to establish subject baseline and appropriateness to proceed. Heparin is administered for anticoagulation once access is complete. All iCMR procedures were performed with the patient at steady-state (in room air—21% oxygen) under general anesthesia in Zone 4 (actual magnet room) from which all ferromagnetic objects must be excluded [3]. All monitoring is MR-conditional throughout this procedure.

### Study methods

Subjects with CHD underwent real-time 1.5 T iCMR between March, 29th 2018 to May, 2nd 2019. A recently developed novel passive catheter tracking technique using a real-time single-shot balanced steady-state free precession (bSSFP) with flip angle (FA) 35°, echo time (TE) 1.3 ms, repetition time (TR) 2.7 ms, and a non-selective partial saturation (pSAT) pre-pulse [3, 6] was used to visualize the gadolinium-filled balloon, MR-conditional guidewire, and cardiac structures simultaneously to aid in completion of right heart catheterization (RHC) and LHC/aortic pull back under real-time iCMR visualization [3].

Phase contrast CMR (PC-CMR) was used to measure Qp and Qs blood flow. PC images were acquired over 40 cardiac phases, TE/TR = 2.7/4.4 ms, with two signal averages during free breathing, 2 × 2 × 8 mm resolution, SENSE acceleration factor = 2, with the velocity encoding gradient set to 25% above the expected maximum velocity in each vessel. Vendor-provided background phase correction was used, and post-processing was performed using cvi42 (Circle Cardiovascular Imaging Inc., Calgary, Alberta, Canada).

**Fig. 2:**
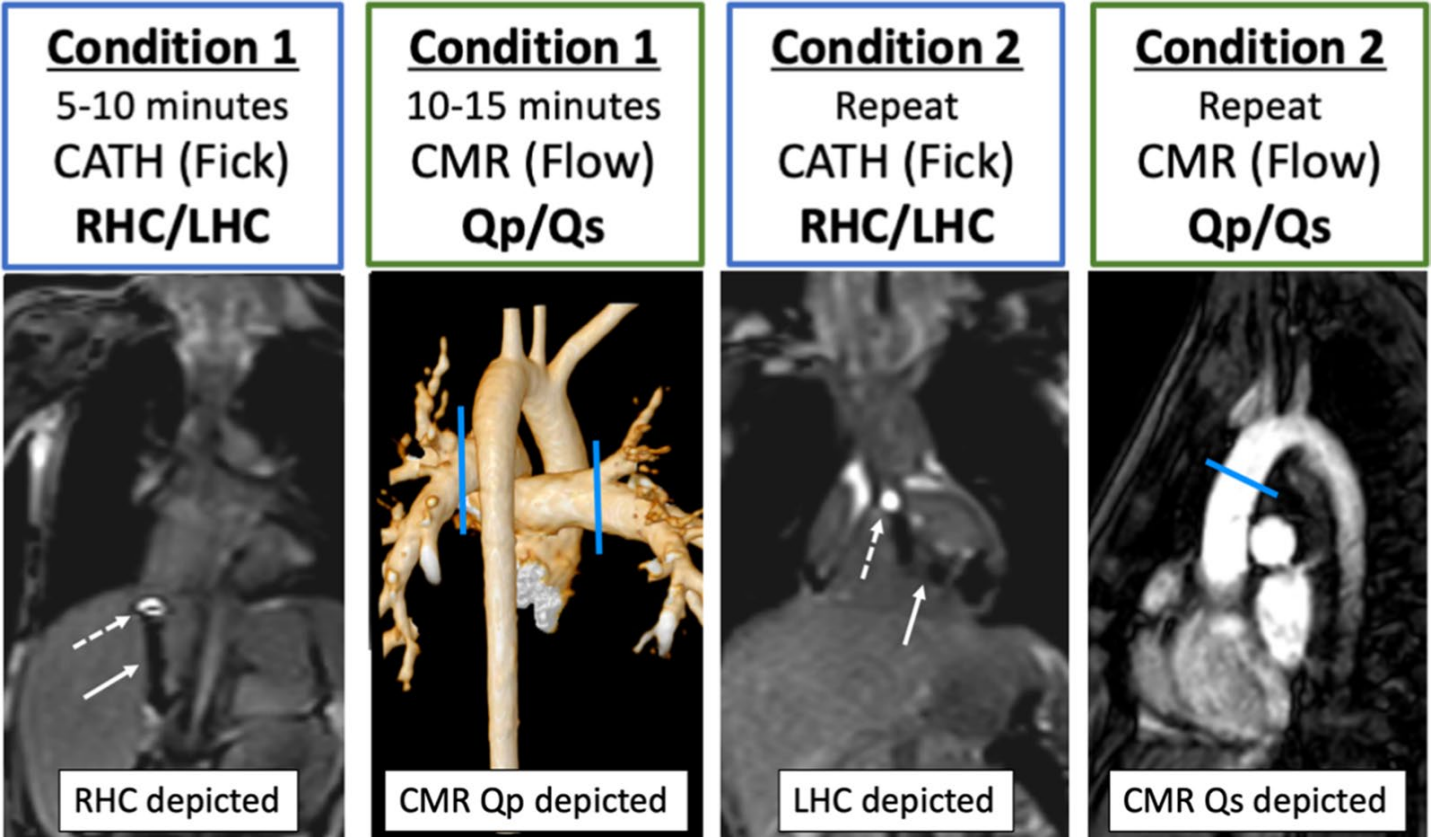
Test–retest catheter-based Fick and CMR-based flow reliability. A series of two conditions were performed to evaluate intra- and inter-rater reliability between catheter-based Fick and CMR-derived flow hemodynamics. The first condition was baseline catheter-based Fick (right heart catheterization (RHC)/left heart catherization (LHC)) and CMR-based flow (pulmonic flow (Qp)/systemic flow (Qs)) measurements. The second condition was repeat measurement under the same conditions (Cath: RHC/LHC + CMR: Qp/Qs flows). Dashed white arrow – Gadolinium-filled balloon; Solid white arrow – MR-conditional guidewire; Blue line –CMR flow vector

A series of two conditions were performed while the patient was mechanically ventilated on room air to evaluate intra- and inter-rater reliability (Fig. 2). The first condition (C1) was baseline catheter-based Fick and CMR-based arterial flow patterns using PC-CMR. The second condition (C2) is performed under the same clinical settings with no change in ventilation, oxygenation, or hemodynamics. Vitals were monitored and a blood gas was drawn between conditions to ensure steady state of the subject. Extreme care was taken to keep the total fluid status the same during C1 and C2. Total flush for catheters was very minimal (total ~ 10–15 ml) between sampling measurements. The same VO_2_ and blood hemoglobin is used for both conditions. Depending on the subject’s anatomy and physiology the saturations and pressures obtained differed slightly. In general, we obtained saturations in the conduit, superior vena cava, branch PAs, left atrium (LA)/PVs, and femoral artery. Pressures were obtained in the inferior vena cava (IVC)/superior vena cava (SVC), hepatic vein with wedge pressures, conduit, branch PA with wedge pressures, right atrium (RA), LA, right ventricle (RV), left ventricle (LV), ascending aorta, descending aorta, and femoral artery. For example, a patient with Fontan completion would have pressure/saturation measurements obtained in the IVC, conduit, SVC, branch PAs, and femoral artery. If a Fontan patient had a patent fenestration then we would attempt to obtain saturations in the common atrium/PVs as well. Similar to standard catheterization lab practices, single sample measurements were obtained in C1 and repeated again in C2. Average time between the start of C1 and the end of C2 was under thirty minutes.

### Statistical considerations

The Pearson correlation coefficient (PCC) is used to measure test–retest reliability and the concordance correlation coefficient (CCC) is used to quantify agreement between catheter-based Fick and CMR-based flow measurements [7]. Descriptive analyses of the continuous/categorical data were performed using means, confidence intervals, proportions and frequencies. This data was displayed on Bland–Altman and scatter plots (GraphPad Prism, version 8.0.0, GraphPad Software, San Diego, California, USA).

## Results

### Patient demographics

**Fig. 3:**
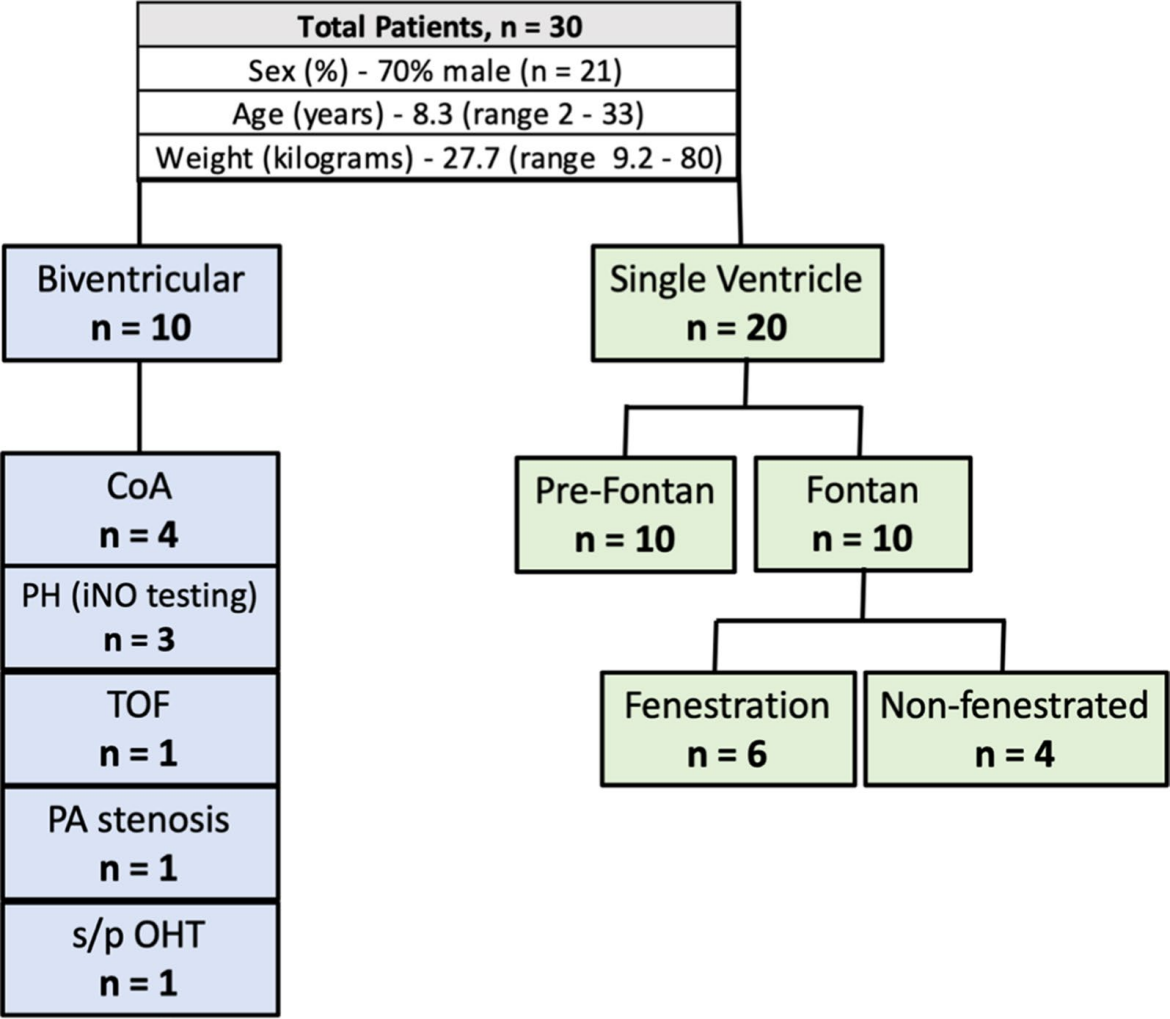
Basic subject demographics. (CoA = Coarctation of the aorta; PH = Pulmonary hypertension; iNO = Inhaled Nitric Oxide; TOF = tetralogy of Fallot; PA = Pulmonary artery; OHT = Orthotopic heart transplantation)

Thirty CHD (21 male) subjects participated in the iCMR reproducibility study at our institution (Fig. 3). Median age and weight were 8.3 years and 27.7 kg (range: 2–33 yrs and 9.2–80 kgs), respectively. Twenty subjects had single ventricle anatomy with 10 pre-Fontan and 10 post-Fontan evaluations for protein losing enteropathy (PLE) and/or cyanosis. Ten subjects had bi-ventricular (BiV) anatomy, four were referred for coarctation of the aorta (CoA) evaluations, 3 underwent vaso-reactivity testing with inhaled nitric oxide (iNO), one had multiple cardiac shunts, one underwent branch PA stenosis evaluation, and the remaining subject was status post heart transplant.

### Procedural results

**Fig. 4:**
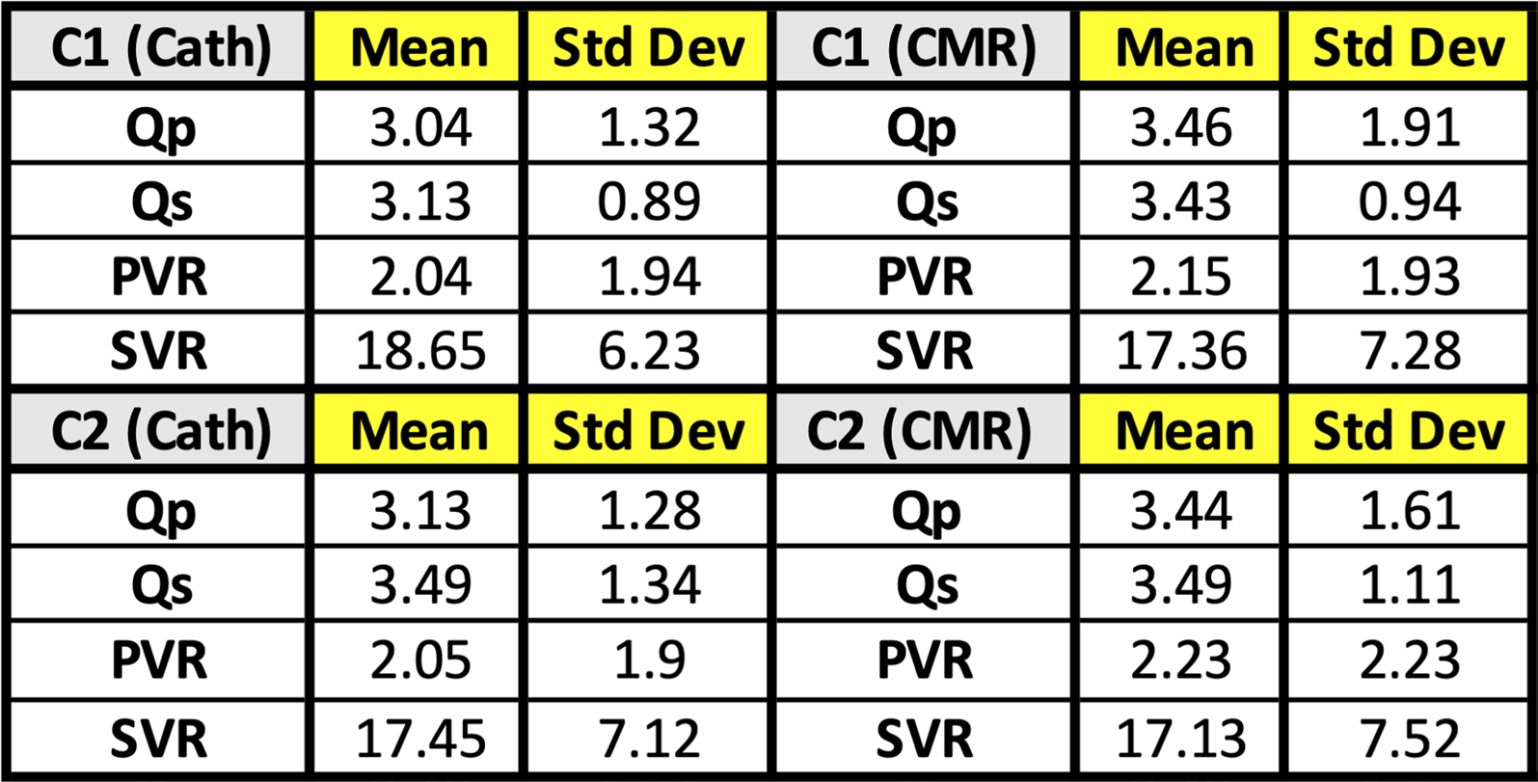
Summary of basic iCMR hemodynamics. Comparison of mean and standard deviation (std dev) measurements for catheterization and CMR hemodynamics for condition 1 (C1) and condition 2 (C2). *Qp* = *Pulmonary blood flow; Qs* = *Systemic blood flow*

Real-time iCMR-guided RHC (30/30 subjects, 100%), retrograde and prograde LHC/aortic pull back (30/30 subjects, 100%) were successfully performed. Total success rate to obtain required data points to complete Fick principle calculations for all patients was 321/328 (98%). No catheter related complications were encountered. Average time taken for first pass RHC and LHC/aortic pull back was 5.1, and 2.9 min, respectively. One patient with multiple shunts (atrial septal defect, ventricular septal defect, and patent ductus arteriosus) who was referred for vaso-reactivity testing was deemed to be an outlier and excluded from the study’s analysis of PCC, CCC, and coefficient of determination. Comparison of invasive catheterization versus iCMR hemodynamic measurements mean and standard deviations are outlined in Fig. 4.

### Test–retest reliability

**Fig. 5:**
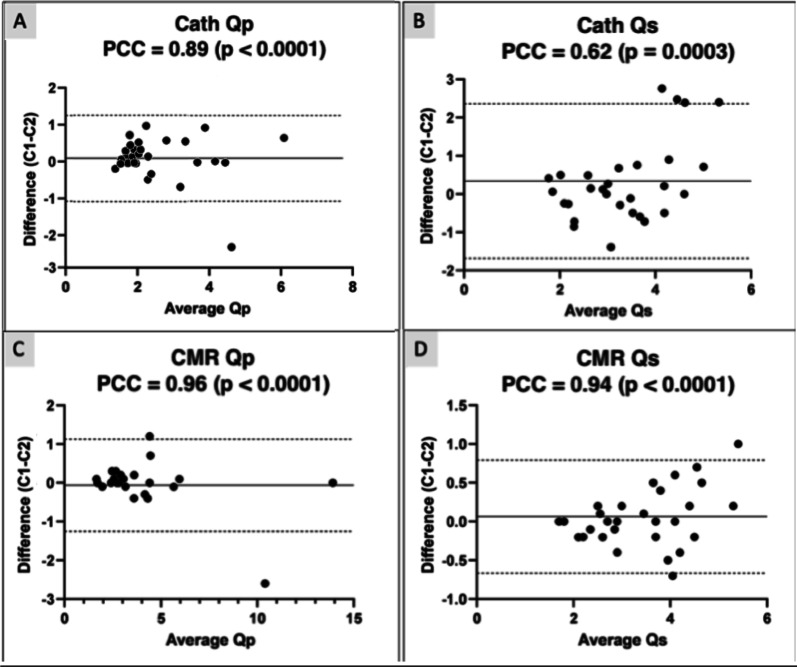
iCMR intra-rater reliability testing. Bland–Altman plots depicting Pearson correlation coefficients (PCC) to measure test–retest reliability testing between conditions 1 (C1) and 2 (C2) for catheter-based Fick hemodynamics for (**A**) Qp (**B**) Qs and CMR-derived flow hemodynamics for (**C**) Qp and (**D**) Qs*. Qp* = *Pulmonary blood flow; Qs* = *Systemic blood flow*

The PCC for variables measured are shown in Fig. 5 with a Bland-Atlman plot. The PCC for catheter-derived Qp (0.89, p < 0.001) is lower than CMR-derived Qp (0.96, p < 0.001). The PCC for catheter derived Qs (0.62, p < 0.001) was considerably lower than CMR derived Qs (0.94, p < 0.001).

### Cath and CMR agreement

**Fig. 6:**
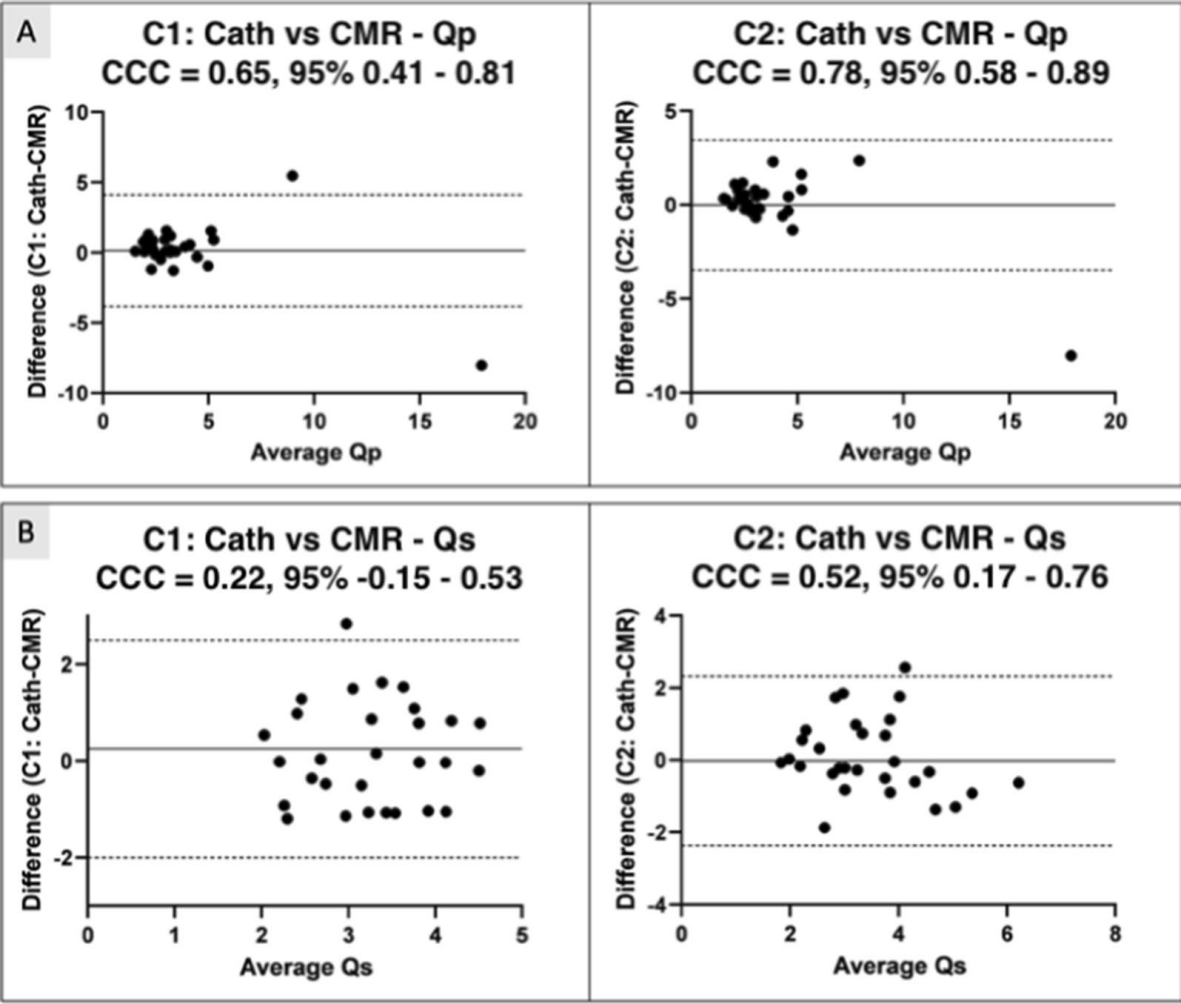
iCMR inter-rater reliability testing. Bland–Altman plots depicting concordance correlation coefficient (CCC) to measure agreement between catheter-based Fick and CMR-derived flow hemodynamics in condition 1 (C1) and condition 2 (C2) for (**A**) Qp and (**B**) Qs. *Qp* = *Pulmonary blood flow; Qs* = *Systemic blood flow*

Figure 6 is a Bland-Atlman plot of the CCC results. There was fair agreement (CCC > 0.8) for Qp between catheterization and CMR measurements at baseline (C1-CCC = 0.65, 95% CI 0.41–0.81), and retested conditions (C2-CCC = 0.78, 95% CI 0.58–0.89).

### iCMR scatter plot

**Fig. 7:**
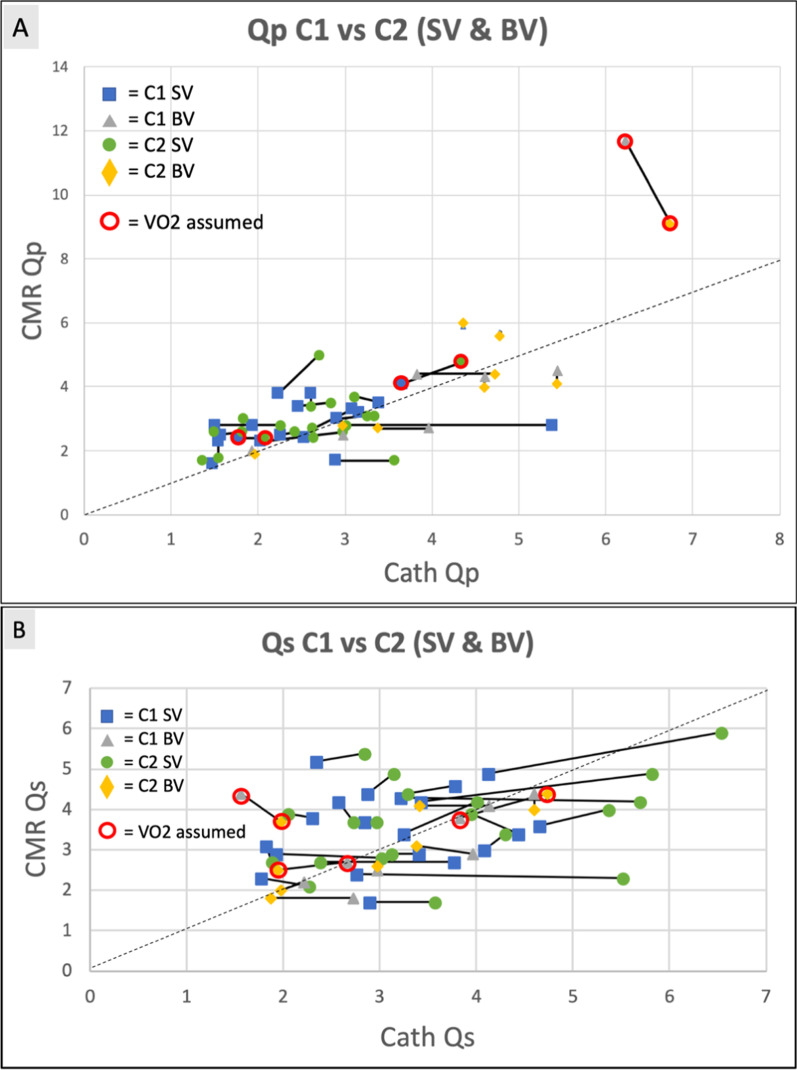
iCMR flow scatter plots. Comparison of Cath vs CMR blood flow measurements for (**A**) Qp and (**B**) Qs. Subjects are grouped based on their underlying anatomy (single ventricle and biventricular). C1 and C2 for each subject is connected by a black line. The dashed line represents an ideal linear relationship. The subjects where VO2 was assumed are outlined in red. *Qp* = *Pulmonary blood flow; Qs* = *Systemic blood flow; SV* = *Single ventricle; BV* = *Biventricular; C1* = *Condition 1; C2* = *Condition 2; VO2* = *Oxygen consumption*

Figure 7 depicts Qp and Qs by graphing an iCMR scatter plot with cath Fick calculations on the x-axis and CMR flows on the y-axis. In addition, C1 and C2 as well as single ventricle and biventricular patients are separated to further illustrate the potential differences in each patient population. C1 and C2 for each subject is connected with a black line. The patient’s with assumed VO2 were outlined in red to help reduce any confounding variables.

### Coefficient of determination

Catheterization, CMR, and comparisons were summarized in Table 1 with coefficients of determination. Qs coefficient of determination for CMR-based flows (R^2^ = 0.88) was found to be much higher than the catheter-derived method (R^2^ = 0.39).

**Table 1:** iCMR coefficient of determination

Linear regression model n = 29								
	X = Cath C1		X = CMR C1		X = CMR C1		X = CMR C2	
	Y = Cath C2		Y = CMR C2		Y = Cath C1		Y = Cath C2	
	*Y* = slope*x + intercept	*R*^*2*^	*Y* = slope*x + intercept	*R*^2^	*Y* = slope*x + intercept	*R*^2^	*Y* = slope*x + intercept	*R*^2^
Q_p_	0.86x + 0.51	0.80	0.79x + 0.69	0.92	0.50x + 1.32	0.51	0.65x + 0.36	0.65
Q_s_	0.94x + 0.54	0.39	1.09x− 0.23	0.88	0.22x + 2.39	0.05	0.62x + 1.34	0.28

Overall coefficient of determination using a linear regression model is calculated between Cath, CMR, and the two method for C1 and C2

Qp, Pulmonary blood flow; Qs, Systemic blood flow; Condition 1; C2, Condition 2

## Discussion

In this novel iCMR study, we compared and assessed real-time catheter-based Fick principle to CMR-derived flow measurements. Test–retest PCC reliability results for CMR were similar for all variables (Qp, Qs; p < 0.001). However, test–retest PCC reliability results for catheterization were similar only for Qp (p < 0.001), whereas findings were lower for Qs (p = 0.003). CMR-derived Qs is significantly more dependable than the catheter-based method. This finding can have significant consequences in a clinician’s ability to properly treat and counsel patients. Interestingly, there is good agreement between catheterization and CMR methods to determine PVR which is critically important for the single ventricle population. CMR alone is not able to determine PVR due to the inability to measure pressure non-invasively, with current standard techniques. iCMR PVR is calculated by using the transpulmonary gradient from direct catheter measurements during the same preload and afterload conditions. In an effort to further reduce radiation exposure, iCMR is emerging as a powerful modality in the assessment of complex CHD.

Previous studies have attempted to compare catheter and CMR-based hemodynamics, however, methods have been limited given the inability to measure right and left sided hemodynamics at the same time under the same conditions [8–12]. In some cases, hemodynamic comparisons between catheterization and CMR were performed months apart. One study specifically looked at subjects with a Glenn circulation (n = 30) who underwent catheterization and CMR (XMR) under the same anesthesia [13]. The authors concluded that catheter-derived Fick measurements are generally unreliable in patients with a Glenn circulation. They noted poor correlation when comparing catheter-based Fick principle to the CMR-based flow method (Qp-ρc = 0.22; Qs-ρc = 0.24). Fick consistently underestimated *Q*p and overestimated PVR when compared to CMR. In contrast, Fick calculations of *Q*s overestimate CMR-measured *Q*s. Another single center study [14] compared these modalities for pre-Glenn and pre-Fontan patients who had a catheterization and CMR < 1 month apart (n = 26). They noted that measurements between the two modalities should not be used interchangeably (Qp: *r* = 0.04, *p* = 0.86; Qs: *r* = 0.44, *p* = 0.02), with potential clinical significance in estimating PVR.

Another important study by Rogers et al. [15] successfully recruited 102 patients for real-time CMR fluoroscopy guided RHC. They showed high rates of procedural success, excellent safety outcomes, and compared cardiac output and PVR quantifications between CMR and cath. However, a major limitation was the inability to perform intra- and/or inter-rater reliability due to the lack of repeatability in their procedural protocol. Our study focused on evaluating the reproducibility of catheter derived Fick calculations versus CMR based flow calculations and assessing the agreement between the two modalities in patients under the same preload and afterload conditions and with all measurements obtained within 30 min from first sample collection. We showed good CCC (> 0.8) implying a good agreement between catheter-based Fick and CMR-based flow measurements for Qp at baseline (C1) and retested (C2) conditions. With the new FDA-cleared MR-conditional guidewire, we were also able to reliably perform a diagnostic LHC within the CMR-magnet. However, we noted there was poor agreement between the two modalities for Qs assessment at baseline (C1) and retested (C2) conditions. iCMR enables clinicians to determine a more thoughtful and accurate hemodynamic evaluation in the CHD population. Additional studies are needed to correlate findings between single ventricle and BiV patients in the iCMR environment including effect of aortopulmonary collaterals burden on Qp, Qs, PVR, and SVR.

Determining PVR remains critically important to risk stratify single ventricle patients for possible catheter and/or surgical interventions. Eligibility for single ventricle palliation is largely determined by direct catheter-based pressure measurements. CMR remains limited by the inability to directly measure vessel and chamber pressures. iCMR is a step toward a more thorough understanding of a patient’s true hemodynamics. Our institution has established a set criteria for pre-Glenn patients where CMR alone is appropriate to proceed with single ventricle palliation [16]. Given the ongoing debate for optimal pre-surgical screening for single ventricle palliation, iCMR evaluations will allow clinicians to continue to grow in their understanding of the overlapping utility of this combined iCMR modality.

## Limitations

Our study limitations include a relatively small sample size and combining single ventricle/biventricular subjects, however, the aim of this study was to better understand the test–retest reliability of catheter-based Fick and CMR-based flow measurements across CHD physiologies under the same physiological conditions at the same time. Another potential limitation of the iCMR procedure is the inconsistent visualization of the gadolinium-filled balloon catheter. This issue has been improved by the use of a real-time pSAT sequence described by Velasco Forte et al. [6]. This sequence has been adapted for simultaneous visualization of the gadolinium-filled balloon, MR-conditional guidewire, and cardiac structures as described by Veeram Reddy et al. [3]. In addition, iCMR continues to be extremely limited by the available MR-safe and/or compatible equipment. It is often difficult to enter small and/or stenotic vessels in the CHD population due to the lack of versatility in equipment. It is our hope that industry will be encouraged to fill the obvious device gap in the iCMR arena. Without the manufacturing of a more diverse set of wires and catheters, the field will continue to be limited to routine diagnostics and simple interventions.

Furthermore, the single ventricle population is uniquely positioned to benefit from this modality. We are performing Fontan fenestration test occlusions within the iCMR environment. An iCMR evaluation provides a more critical evaluation of Fontan pressures and Qs at the time of Fontan fenestration test occlusion before referral for fenestration device closure [3, 17]. By using accurate Qs flow measurements with simultaneous catheter-based pressure measurements, the clinician will become more confident to make an informed decision for Fontan fenestration device closure.

In addition, CMR provides precise anatomical and functional data to plan complex CHD interventions [18, 19]. In recent years, congenital cardiology has been investigating an exponential increase in the indications for CMR to answer significant clinical questions that are not able to be assessed by other imaging modalities alone. We continue to investigate non-invasive measurements of blood oxygen saturation, PVR, and APC burden. The iCMR environment allows for a more critical evaluation of complex CHD subjects by allowing for assessment of transcatheter pressures during CMR derived flow measurements.

## Conclusion

Real-time, radiation-free iCMR-guided cardiovascular catheterizations allows for simultaneous measurement of catheter-based hemodynamics and CMR-derived flows to quantify and compare important hemodynamic variables including Qp and Qs. Most notably, CMR-derived flows have higher test–retest reliability for Qs when compared to catheter-based Fick principle. iCMR evaluations allow for more reproducible hemodynamic assessments in the CHD population. This pilot study further endorses non-invasive diagnostic parameters to better serve the CHD population in the planning stages prior to catheter-based and/or surgical interventions.

## Data Availability

The datasets used and/or analyzed during the current study are available from the corresponding author on reasonable request.

## Abbreviations

BiV: Biventricular
bSSFP: Balanced steady state free precession
CHD: Congenital heart disease
CMR: Cardiovascular magnetic resonance
CoA: Coarctation of the aorta
FDA: United States Food and Drug Administration
iCMR: Invasive cardiovascular magnetic resonance
iNO: Inhaled nitric oxide
IVC: Inferior vena cava
LA: Left atrium/left atrial
LHC: Left heart catheterization
LV: Left ventricle/left ventricular
MV: Mixed venous
PA: Pulmonary artery
PC: Phase contrast
PCC: Pearson correlation coefficient
PH: Pulmonary hypertension
pSAT: Partial saturation
PV: Pulmonary vein
PVR: Pulmonary vascular resistance
Qp: Pulmonary blood flow
Qs: Systemic blood flow
RA: Right atrium/right atrial
RHC: Right heart catheterization
RV: Right ventricle/right ventricular
SVC: Superior vena cava
SVR: Systemic vascular resistance
TOF: Tetralogy of Fallot
TPG: Transpulmonary pressure gradient
TSG: Transsystemic gradient
VO2: Oxygen consumption
